# Acute liver injury induces expression of FGF23 in hepatocytes via orphan nuclear receptor ERRγ signaling

**DOI:** 10.1016/j.gendis.2022.06.003

**Published:** 2022-07-01

**Authors:** Yoon Seok Jung, Yong-Hoon Kim, Kamalakannan Radhakrishnan, Jung-Ran Noh, Jung Hyeon Choi, Hyo-Jin Kim, Jae-Ho Jeong, Steven Dooley, Chul-Ho Lee, Hueng-Sik Choi

**Affiliations:** a School of Biological Sciences and Technology, Chonnam National University, Gwangju 61186, Republic of Korea; b Laboratory Animal Resource Center, Korea Research Institute of Bioscience and Biotechnology, 125 Gwahak-ro, Yuseong-gu, Daejeon 34141, Republic of Korea; c Department of Functional Genomics, KRIBB School of Bioscience, Korea University of Science and Technology (UST), Daejeon 34113, Republic of Korea; d Combinatorial Tumor Immunotherapy MRC, Chonnam National University Medical School, Hwasun-gun, Jeonnam 58128, Republic of Korea; e Department of Microbiology, Chonnam National University Medical School, Gwangju 61186, Republic of Korea; f Molecular Hepatology Section, Medical Faculty Mannheim, Heidelberg University, 69117 Heidelberg, Germany

Fibroblast growth factor 23 (FGF23) is an osteocyte- and osteoblast-derived hormone that primarily regulates phosphate and vitamin D metabolism. Circulatory FGF23 levels are abnormally increased in pathological conditions like acute or chronic kidney injury, resulting in disease progression as well as increased rates of morbidity and mortality.^1^ However, FGF23 production in acute liver injury is not fully investigated. In this study, we report that carbon tetrachloride (CCl_4_)-induced acute liver injury upregulates hepatic estrogen-related receptor gamma (*ERRγ*) and *FGF23* gene expression and FGF23 secretion from liver. Hepatocyte specific depletion of *ERRγ* blunted CCl_4_-induced hepatic *FGF23* promoter activity, *FGF23* gene expression and FGF23 levels. Further, treatment with ERRγ-specific inverse agonist GSK5182 also efficiently inhibited CCl_4_-induced acute liver injury-mediated hepatic *FGF23* gene expression and circulatory FGF23 levels *in vivo*. Taken together, these results firstly describe a detailed molecular mechanism of hepatic *FGF23* gene expression induction in an acute liver injury condition. Further, we present evidence that inhibiting *ERRγ* transactivation by the small molecule GSK5182 may be a useful strategy to control the devastating circulatory levels of FGF23.

A previous study suggested that FGF23 production is regulated by JAK/STAT signaling in mice liver.^2^ However, mechanism associated with *FGF23* gene expression in liver disease are still unknown. In this context, we intraperitoneally injected WT mice with CCl_4_ (1 mL/kg body weight of 10% CCl_4_ dissolved in corn oil) for 6 h and assessed messenger RNA (mRNA) expression in major tissues including brain, heart, lung, liver, spleen, kidney and bone. *ERRγ* and *FGF23* mRNA expressions were specifically increased in liver, but not in other major organs (Fig. 1A, B). To determine the expression pattern of CCl_4_-induced *FGF23* gene expression, we injected mice with CCl_4_ for different time periods. Hematoxylin and eosin (H&E) staining of mouse liver samples showed that acute liver started to occur at the 3 h time point upon CCl_4_ injection, and kept worsening at 6, 12 and 24 h (Fig. S1A). *FGF23* mRNA expression, protein expression and plasma intact FGF23 levels started to increase from 3 h on, reaching a peak after 6 h of CCl_4_ injection (Fig. S1B-D). Taken together, these results suggested that CCl_4_ induced *FGF23* gene expression and secretion in mouse liver.

**Figure 1:**
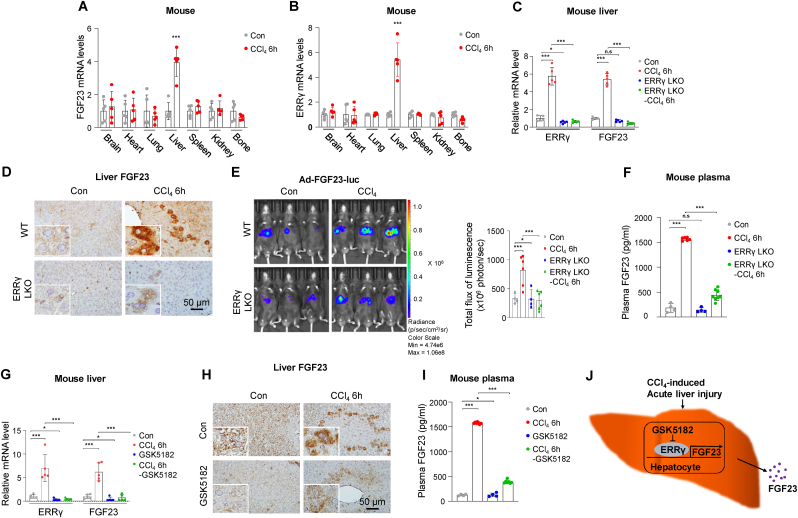
CCl_4_-induced acute liver injury increases *FGF23* gene expression and secretion in mouse liver through *ERRγ.***(A, B)** Quantitative PCR analysis of total RNA obtained from the livers of mice injected with CCl_4_ (1 mL/kg body weight of 10% CCl_4_ dissolved in corn oil) for 6 h (*n* = 5 per group). **(C–F)** WT and *ERRγ*-LKO mice were injected with CCl_4_ for 6 h (*n* = 5 per groups). **(C)** Quantitative PCR analysis of total RNA isolated from livers. **(D)** Representative images of FGF23 immunohistochemical analysis in liver sections. **(E)** Representative *in vivo* images of hepatic *FGF23* promoter WT-luciferase (Ad-*FGF23*-luc) activity in WT and *ERRγ*-LKO mice injected with or without CCl_4_ (*n* = 4 for WT-Con and *ERRγ*-LKO Con; *n* = 6 for WT-CCl_4_ and *ERRγ*-LKO CCl_4_ group). **(F)** Plasma FGF23 levels measured by ELISA. **(G**–**I)** WT mice were injected with CCl_4_ in the presence or the absence of GSK5182 and sacrificed after 6 h (*n* = 5 per group). **(G)** Quantitative PCR analysis of total RNA isolated from liver. **(H)** Representative images of FGF23 immunohistochemical analysis in liver sections. **(I)** Plasma *FGF23* levels measured by ELISA. **(J)** Schematic diagram of *ERRγ*-mediated hepatic *FGF23* gene expression and secretion in CCl_4_-induced acute liver injury. Data indicate mean ± SEM values. Data in (A) and (B) were analyzed by two-tailed Student's *t* test. Data in **C, E, F, G** and **I** were analyzed by ordinary one-way ANOVA with Tukey's multiple comparisons test. Significance levels denoted as ∗*P* < 0.05; ∗∗∗*P* < 0.001; *n.s*., not significant.

CCl_4_ injection possibly leads to nephrotoxicity and acute/chronic kidney injury results in increased production of renal FGF23. We investigated the gene expression in our model of CCl_4_-acute liver injury. The levels of pro-inflammatory cytokine interleukin-6 *(IL6)* mRNA were found to be increased in liver but not in kidney tissues in response to CCl_4_ injection (Fig. S2A). Alanine aminotransferase (ALT) and aspartate aminotransferase (AST) levels, which are markers of liver injury, were significantly increased in the plasma of CCl_4_-injected mice (Fig. S2B, C). H&E staining of mouse liver and kidney samples showed that acute liver injury was induced by CCl_4_ injection without causing any damage to kidney (Fig. S2D). The kidney injury markers blood urea nitrogen (BUN) and creatinine remained unchanged between control and CCl_4_-injected groups (Fig. S2E, F). These results indicated that CCl_4_-acute liver injury increased *FGF23* gene expression without causing renal damage.

We previously reported that *FGF23* gene expression is transcriptionally regulated in the liver by *ERRγ* in response to folic acid-induced acute kidney injury (FA-AKI).^3^ We also found that CCl_4_-acute liver injury resulted in increased hepatic *ERRγ* gene expression. To investigate whether *ERRγ* was the upstream regulator of hepatic *FGF23* gene expression and secretion in our current study, we employed hepatocyte specific *ERRγ* knockout mice (*ERRγ*-LKO) and intraperitoneally injected with CCl_4_ for 6 h. The expression of hepatic *ERRγ* and *FGF23* mRNA was increased in CCl_4_-injected WT mice, but was significantly inhibited in *ERRγ*-LKO mice (Fig. 1C). CCl_4_-mediated induction of hepatic FGF23 protein expression and plasma FGF23 levels was significantly blunted by hepatic loss of *ERRγ* expression (Fig. 1D, F).

To elucidate the molecular mechanism underlying *ERRγ*-regulated *FGF23* gene expression in response to CCl_4_-induced acute liver injury, we utilized mouse *FGF23* promoter luciferase construct fused with adenovirus (Ad-*FGF23*-luc). WT and *ERRγ*-LKO mice were injected with Ad-*FGF23*-luc via tail vein and mice were treated 3 days post-injection with CCl_4_ for 6 h. *In vivo* imaging showed that hepatic *FGF23* promoter activity was significantly increased in CCl_4_ injected mice compared with control mice. However, no difference was detected in hepatic *FGF23* promoter activity between control and CCl_4_-injected *ERRγ*-LKO mice (Fig. 1E). This result showed that ERRγ directly binds with *FGF23* promoter to transcriptionally regulate hepatic *FGF23* gene expression in response to CCl_4_-acute liver injury. Then, we measured FGF23 levels in the plasma of WT and *ERRγ*-LKO mice treated with or without CCl_4_. Altogether, these results suggest that hepatic *ERRγ* expression was required to transcriptionally regulate hepatic *FGF23* gene expression in CCl_4_-induced acute liver injury.

Finally, we tested the pharmacological inhibition of ERRγ using inverse agonist GSK5182 to inhibit *FGF23* gene expression in response to CCl_4_ injection. GSK5182 is an *ERRγ*-specific inverse agonist, which inhibits the transcriptional activity of *ERRγ* and thereby decreases the expression of *ERRγ* target genes. WT mice were injected with or without CCl_4_ in the presence or the absence of GSK5182. We found that GSK5182 treatment significantly inhibited CCl_4_-induced hepatic *ERRγ* and *FGF23* mRNA expression in WT mice (Fig. 1G). Further, CCl_4_-induced hepatic FGF23 protein expression and plasma FGF23 levels were also inhibited by GSK5182 based on the results of immunohistochemical analysis and ELISA, respectively (Fig. 1H, I). These results suggest that inverse agonist-mediated inactivation of *ERRγ* transactivation significantly inhibits CCl_4_-mediated hepatic *FGF23* gene expression.

Hepatic *FGF23* production was elevated in autosomal dominant polycystic kidney disease and childhood biliary atresia.^4^ We previously reported FA-AKI induced *FGF23* gene expression and secretion by hepatocytes.^3^ AKI upregulated *IL6* in kidney, which mediated organ-to-organ communication to induce haptic *FGF23* production via *ERRγ*. Here, we showed that CCl_4_-acute liver injury upregulates hepatic *FGF23* synthesis via *ERRγ* with significant increase in hepatic *IL6* expression. Pro-inflammatory cytokine *IL6* is the common factor in both FA-AKI and CCl_4_-acute liver injury and *IL6* induces *ERRγ* gene expression in liver. In line with our data, Kumar et al recently reported upregulation of FGF23 production in total liver as response to CCl_4_ hepatotoxicity, diet-induced fatty liver, and bile-acid-induced cholestatic liver disease.^5^ They show *in vitro* that lipopolysaccharide mediated toll-like receptor (TLR) 4 signaling induced FGF23 production indirectly in macrophages, together with IL1β and TNFα in combination, and TLR2 agonist Pam2CSK3. The liver resident macrophages or Kupffer cells, but not the hepatocytes or hepatic stellate cells, were found as source of FGF23. They, however, did not dissect the cell types of FGF23 expression in the more general disease settings CCl_4_ treatment, high-fat diet feeding or bile duct ligation. In the current study, we report that hepatocytes are the major source of FGF23 in CCl_4_-induced acute liver injury and confirmed the finding by showing significantly decreased FGF23 production *ERRγ*-LKO mice.

ERRγ is a key regulator of hepatic FGF23 production *in vivo* in response to CCl_4_-induced acute liver injury (Fig. 1J). Hepatocyte-specific ablation of *ERRγ* expression or inverse agonist-mediated inhibition of *ERRγ* transcriptional activity significantly blunted CCl_4_-induced hepatic FGF23 synthesis. Therefore, we suggest that blocking the ERRγ signaling is an attractive strategy to reduce pathologically abnormal circulatory FGF23 levels in acute liver injury.

## Ethics declaration

All animal procedures were approved by the Institutional Animal Care and Use Committee of KRIBB (KRIBB-AEC-20135). All animal experiments were performed in accordance with the Guide for the Care and Use of Laboratory Animals published by the US National Institutes of Health.

## Author contributions

Y.S.J., Y.-H.K., C.H.L., and H.-S.C. designed research. Y.S.J., Y.-H.K., H.-J.K., J.-R.N., and J.H.C. performed the experiments. Y.S.J., Y.-H.K., K.R., J.-H.J., and S.D. analyzed and interpreted the results. Y.S.J., Y.-H.K., K.R., and H.-S.C. drafted and revised the manuscript. All authors reviewed and agreed with the manuscript content.

## Conflict of interests

Authors declare no conflict of interests.

## Funding

This work was supported by the National Research Foundation (NRF) basic science research program Korean government (Ministry of Science and ICT), Republic of Korea (No. 2020R1A6A3A01096145, 2020) (No. NRF-2019R1C1C1005319, 2019) (No. NRF-2017R1A6A3A04006167, 2017) (No. NRF-2020R1A2C3006952, 2020); and (No. NRF-2021R1A2C3004923, 2021). The Federal Ministry of Education and Research-Liver Systems Medicine Program of the Stiftung für Biomedizinische Alkoholforschung, Germany (No. PTJ-031L0043).
