# Supplementary material for: Acute liver injury induces expression of FGF23 in hepatocytes via orphan nuclear receptor ERRγ signaling
Source: Genes Dis. 2022 Jul 1;10(3):679–82. doi: 10.1016/j.gendis.2022.06.003 (PMC10308101; doi:10.1016/j.gendis.2022.06.003)
Supplement: Multimedia component 1 [file mmc1.docx]

**SUPPLEMENTARY DATA**

**MATERIALS AND METHODS**

**Ethics approval**

All animal procedures were approved by the Institutional Animal Care and Use Committee of KRIBB (KRIBB-AEC-20135). All animal experiments were performed in accordance with the Guide for the Care and Use of Laboratory Animals published by the US National Institutes of Health.

**Animal studies**

Eight-week-old male C57BL/6J mice were used for all experiments. C57BL/6J wild-type (WT) mice were obtained from Korea Research Institute of Biosciences and Biotechnology (KRIBB, Daejeon, Korea). C57BL/6J mice containing floxed *ERRγ* exon 2 (*ERRγ* f/f) were obtained from PHENOMIN-iCS, PHENOMIN, the French National Infrastructure in Biology and Health (Illkirch, France). To generate the hepatocyte-specific *ERRγ* knockout line (*ERRγ*-LKO), *ERRγ* f/f animals were crossbred with C57BL/6J-*Alb*-Cre transgenic mice, which express Cre recombinase in hepatocytes under the control of the albumin promoter (Jackson Laboratories, Bar Harbor, ME, USA). Prior to the experiments, mice were acclimatized to a 12-h light/dark cycle at 22 ± 2 °C for 2 weeks with unlimited food and water in a specific pathogen-free facility. WT and *ERRγ*-LKO mice were injected with a single dose of CCl_4_ (1 mL/kg body weight of 10% CCl_4_ dissolved in corn oil) via intraperitoneal injection for the indicated time period. GSK5182 (40 mg/kg in 30% PEG400) was administered intraperitoneally to mice.

**Chemical reagents**

GSK5182 was synthesized at Kyungpook National University (Daegu, Republic of Korea) and DGMIF (Daegu-Gyeongbuk Medical Innovation Foundation, Daegu, Republic of Korea) and dissolved in 30% polyethylene glycol 400 (PEG400, USB, Cleveland, OH, USA). GSK5182 used at a concentration of 40 mg/kg for *in vivo* experiments. CCl4 (Sigma-Aldrich Chemical Co., St Louis, MO, USA) was dissolved in corn oil to a 10% solution and injected as 1 ml/kg of body weight for in vivo experiments.

**Histopathology and immunohistochemical analysis**

Liver samples were fixed in 10% neutral buffered formalin, embedded in paraffin, cut into 5-μm-thick sections and stained with hematoxylin and eosin (H&E). Images were captured using a light microscope (BX51; Olympus Corporation, Tokyo, Japan). Rat monoclonal anti-FGF23 (R&D systems, Clone # 283507, Cat # MAB26291) was used for immunohistochemical staining of FGF23.

**Blood analysis**

Levels of plasma alanine aminotransferase (ALT), aspartate transaminase (AST), blood urea nitrogen (BUN) and creatinine were determined using an automated blood chemistry analyzer (AU480; Beckman Coulter, Krefeld, Germany).

**Recombinant adenoviruses**

Mouse *FGF23* gene promoter was PCR amplified from mouse genomic DNA (Promega) using the following primer: forward, 5′-CGCGGGGCTAGCCAGCAGTCTGCCTTCCAATG- 3′ and reverse, 5′-CGCGGGCTCGAGGAGTGGCTAATGCTGAGTTTG-3′. The adenovirus-expressing mouse *FGF23* promoter (Ad-*FGF23*-luc) was generated using pAdTrack-CMV and pAdEasy-1 system. Virus constructs were purified by cesium chloride density gradient ultracentrifugation. Ad-*FGF23*-luc were used to mice (3 X 10^9^ PFU/mouse) via tail-vein injections.

**RNA isolation and analysis**

Total RNA was isolated using the TRIzol reagent (Invitrogen, CA, USA) according to the manufacturer’s instructions, and used as template to synthesize cDNA with TOPscript RT DryMix (dT18 plus; Enzynomics, Daejeon, Korea). Reverse transcription was performed at 37°C for 5 min, 45°C for 60 min and 95°C for 5 min on a thermocycler (TaKaRa, Shiga, Japan). The cDNAs were analyzed using the Applied Biosystems StepOnePlus real-time PCR system (Applied Biosystems, Foster City, CA, USA) and Power SYBR Green PCR Master Mix (Applied Biosystems). Based on the obtained CT values, the mRNA expression was calculated using the 2^-ΔΔCT^ method. The following primers were used: *ERRγ* (mouse), 5′-AAGATCGACACATTGATTCCAGC-3′ (Forward) and 5′-CATGGTTGAACTGAATTCCCAC-3′ (Reverse); *FGF23* (mouse), 5′-ATGCTAGGGACCTGCCTTAGA-3′ (Forward) and 5′-AGCCAAGCAATGGGGAAGTG-3′ (Reverse); *IL6* (mouse), 5′-CTGCAAGAGACTTCCATCCAG-3′ (Forward) and 5′-AGTGGTATAGACAGGTCTGTTGG-3′ (Reverse). Data were normalized to *L32* (mouse) expression, which was determined using 5′-TCTGGTGAAGCCCAAGATGG-3′ (Forward) and 5′-CTCTGGGTTTCCGCCAGT-3′ (Reverse) primers.

***In vivo* imaging**

C57BL/6J mice were infected with adenovirus fused with mouse *FGF23* promoter-luciferase (Ad-*FGF23*-luc) via tail-vein injection. Mice were treated 3 d post-injection with either CCl_4_ (1 mL/kg body weight of 10% CCl_4_ dissolved in corn oil) or vehicle for 6 h. Mice were imaged using an IVIS Lumina III imaging system (Caliper Life Sciences, MA, USA).

**Measurement of *FGF23* levels**

Plasma-intact FGF23 levels were determined via ELISA, according to the manufacturer’s protocol (Catalog No. 60-6800).

**Statistical analyses**

Data were analyzed with Prism 8 (GraphPad Software, La Jolla, CA, USA) and presented as mean ± SD. Comparison between two groups was performed using the two-tailed Student’s *t*-test, whereas comparison between multiple groups was conducted via ordinary one-way ANOVA with Tukey’s multiple comparison test. Differences were considered statistically significant at *p* < 0.05.

**Supplementary Figure legend**

**Supplementary Figure 1.** CCl_4_-induced acute liver injury increases *FGF23* gene expression and secretion in mouse liver. **(A-C)** WT mice were injected with CCl_4_ for different time intervals as indicated (*n* = 5 per group). **(A)** Representative images of hematoxylin and eosin (H&E) staining in liver sections of control and CCl_4_-injected mice. Arrows indicate vacuolar degeneration of hepatocyte, and arrowheads indicate necrotic area of liver. **(B)** Quantitative PCR analysis of total RNA obtained from liver. **(C)** Representative images of FGF23 immunohistochemical analysis of liver sections. **(D)** Measurement of plasma FGF23 levels by ELISA. Data indicate mean + SEM values. All data were analyzed by two-tailed Student’s *t* test and significance levels denoted as ***p* < 0.01; ****p* < 0.001.

**Supplementary Figure 2.** Acute CCl_4_-toxicity induced acute liver injury without any damage to kidney. **(A-F)** WT mice were injected with CCl_4_ and sacrificed after 6 h (*n* = 5 per group). **(A)** Quantitative PCR analysis of total RNA isolated from mouse liver. Plasma levels of **(B)** alanine aminotransferase (ALT) and **(C)** aspartate aminotransferase (AST) in control and CCl_4_-injected mice. **(D)** Representative images of hematoxylin and eosin (H&E) staining in liver and kidney sections of control and CCl_4_-injected mice. Arrows indicate vacuolar degeneration of hepatocyte. **(E)** Blood urea nitrogen (BUN) and **(F)** creatinine levels in control and CCl_4_-injected mice. Data are expressed as mean + SEM. All data were analyzed by two-tailed Student’s *t* test and significance level indicated by ***p* < 0.01; ****p* < 0.001.
