# Supplementary figures and images for: Acute liver injury induces expression of FGF23 in hepatocytes via orphan nuclear receptor ERRγ signaling
Source: Genes Dis. 2022 Jul 1;10(3):679–82. doi: 10.1016/j.gendis.2022.06.003 (PMC10308101; doi:10.1016/j.gendis.2022.06.003)

## Supplementary Figure 1

**A**

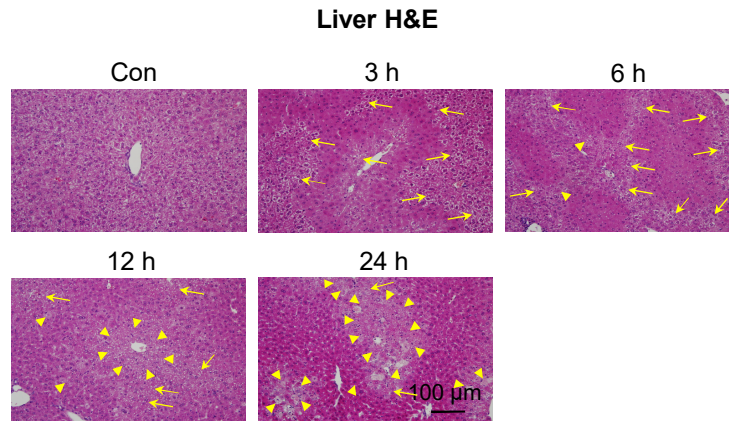

**B**

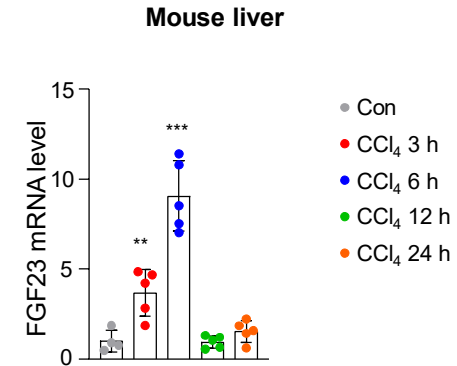

**C**

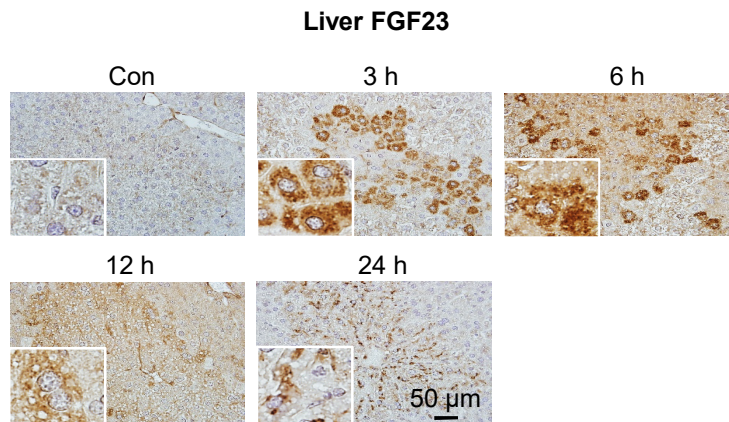

**D**

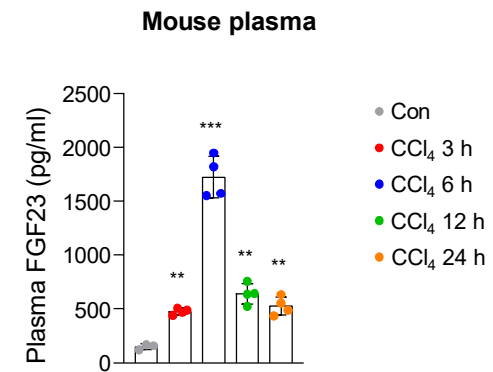

Supplement: Multimedia component 2 [file mmc2.pdf]

## Supplementary Figure 2

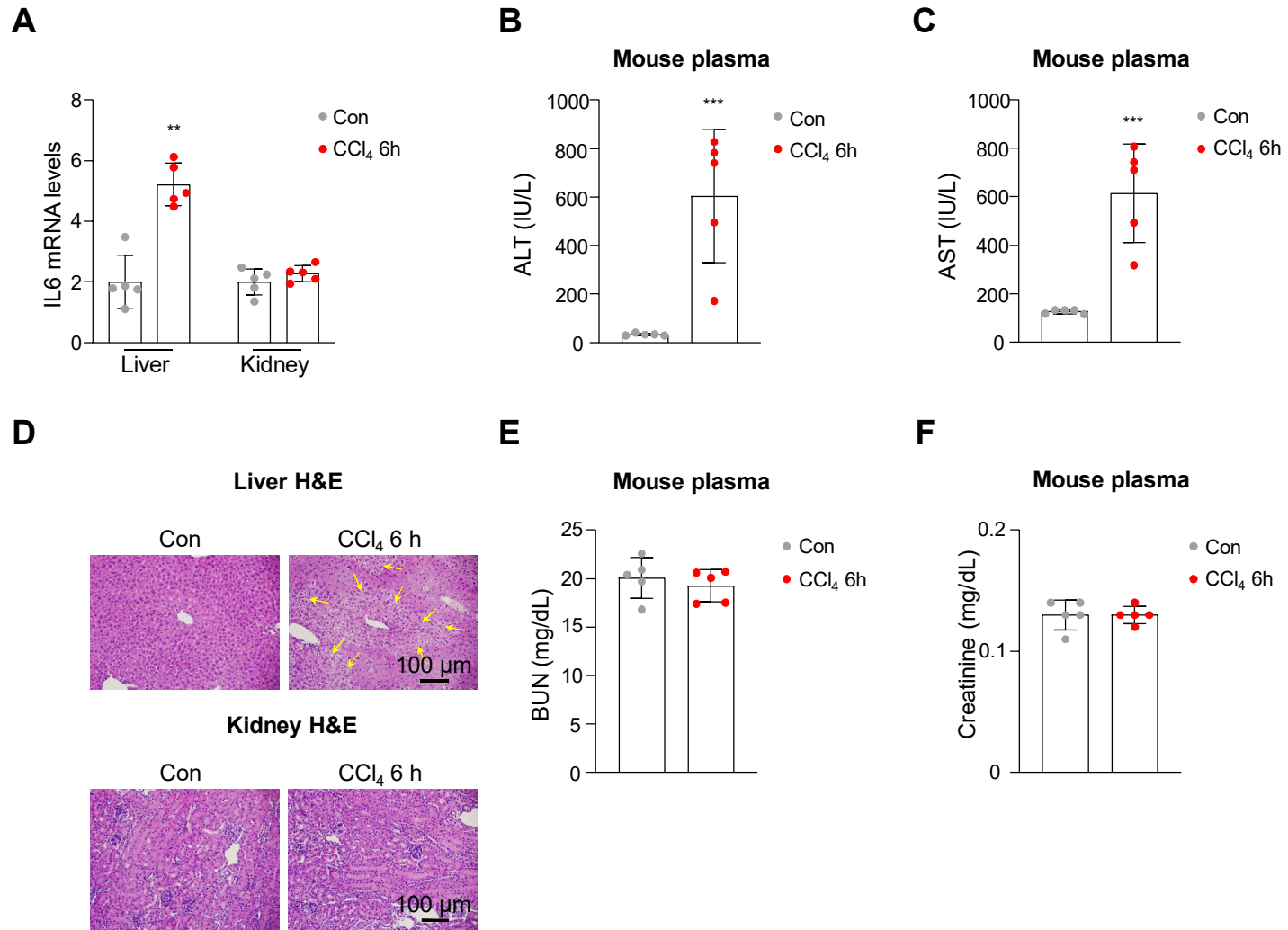

Supplement: Multimedia component 3 [file mmc3.pdf]
